# Impaired myocardial deformation and aortic distensibility by cardiac MRI in girls with Turner syndrome

**DOI:** 10.1038/s41598-024-75312-5

**Published:** 2025-03-12

**Authors:** Nihal M. Batouty, Farah A. Shokeir, Donia M. Sobh, Basma Gadelhak, Wafaa Laimon, Nanees Abdelbadie Salem, Mohamed Abdelghafar Hussein, Ahmed M. Tawfik

**Affiliations:** 1 Department of Diagnostic and Interventional Radiology, Faculty of Medicine, Mansoura University, 12 El-Gomhoreya street, 35112 Mansoura, Egypt; 2 Pediatric Endocrinology and Diabetes Unit, Department of Pediatrics, Faculty of Medicine, Mansoura University Children Hospital, Mansoura University, Mansoura, Egypt; 3 Pediatric department, Faculty of medicine Kafrelsheikh University, Kafrelsheikh, Egypt

**Keywords:** Turner syndrome, Myocardial strain, Cardiac MRI, Aortic distensibility, Aortic strain, Cardiology, Endocrinology

## Abstract

Girls and women with Turner syndrome (TS) suffer from increased risk of cardiovascular diseases. We hypothesized that left ventricular (LV) myocardial strain and aortic elasticity will be impaired in girls with TS. Cardiac MRI of 45 girls with TS and 14 healthy control girls was performed. Tissue tracking was used to assess LV Global Longitudinal (GLS), circumferential (GCS), radial short and long axes (GRS SAX and GRS LAX) in patients compared to controls. Maximal and minimal aortic areas were measured in ascending aorta, proximal descending, and aorta at diaphragm. Regional strain and distensibility were calculated using previously validated formulas. Comparisons were made between patients with and without bicuspid aortic valve (BAV). Inter-observer agreement was assessed for myocardial strain and aortic strain and distensibility. Results of the study showed GLS was significantly impaired in TS patients − 15.6 ± 1.8% compared to the control group − 17.2 ± 1%, p value = 0.013. No significant differences were observed in other strain parameters. Aortic diameter was similar in patients and control groups. Ascending aorta strain and distensibility were significantly lower in TS patients (33 ± 19% and 9.1 ± 5.5 mm Hg^− 1^) compared to control group (55 ± 17% and 13.9 ± 4.9 10^− 3^ mm Hg^− 1^), p values 0.004 and 0.013. No significant differences were found in aortic strain and distensibility in the other 2 regions. No significant differences were observed between TS patients with and without BAV in myocardial strain and aortic strain and distensibility. Substantial to perfect inter-observer agreement was found for myocardial strain and aortic strain and distensibility. Cardiac MRI measurements of LV deformation and aortic function carry potential value as markers for early detection of myocardial and aortic disease in TS patients. Validation of the clinical impact and prognostic role require further longitudinal studies.

**Supplementary Information:**

The online version contains supplementary material available at 10.1038/s41598-024-75312-5.

## Introduction

Turner syndrome (TS) is a chromosomal disorder in females caused by complete or partial absence of the second X-chromosome^1^. Girls and women with TS suffer from lifelong increased risk of cardiovascular disease. Congenital bicuspid aortic valve and aortic coarctation are common. Progressive aortic dilatation may lead to dissection. In addition, other associated cardiometabolic risk factors are common in TS such as arterial hypertension, diabetes, lipid abnormalities, and excess weight^2^.

Cardiac imaging is recommended by the current TS management guidelines as screening tool for cardiovascular abnormalities, and serial follow up of aortic size^3^. However, some TS patients will develop aortic dissection in the absence of significant aortic dilatation^4^. Therefore, measurements of aortic stiffness may be an additional marker to size measurements for prediction of future aortic disease and increased risk of dissection^3,5^.

Left ventricular (LV) myocardial dysfunction may occur in TS patients due to loss of functional elastic properties of the proximal aorta with consequent increased LV afterload^4,6^. LV myocardial dysfunction was also observed in the absence of hypertension and other causes of aortic stiffness, suggesting intrinsic myocardial component of the TS itself^7^.

Cardiac MRI is the standard of care imaging method for assessment of cardiac function and morphology. Myocardial strain analysis by tissue tracking cardiac MRI is a potential adjunct for ejection fraction (EF) in detection of early myocardial dysfunction^8,9^. Compared to speckle tracking echocardiography for strain analysis, cardiac MRI has higher spatial resolution with improved ability to delineate endocardial borders and overcomes Ultrasound limitations such as poor acoustic window degrading the image quality in some patients.

Cardiac MRI also offers great advantage in non-invasive assessment of aortic elasticity at the same examination^10^.

We hypothesized that LV myocardial strain and aortic elasticity will be impaired in girls with TS compared to healthy controls.

## Methods

### Study population

The study was approved by the Institutional review board, Mansoura University, Mansoura, Egypt. This prospective study was performed in accordance to guidelines and regulations of medical research. Participants older than 16 years old, or guardians of younger children gave written informed consent.

From September 2018 till November 2019, consecutive 45 girls with TS were enrolled in the study during routine follow up visits to the Pediatric Endocrinology Clinic at our University Children’s Hospital. Parents of pediatric patients provided written informed consent for participating in the study. Clinical data were collected concurrently with the cardiac MRI scans. All patients were free from cardiac complaints. Exclusion criteria included patients with contraindications to MRI.

Fourteen healthy girls were recruited as a control group from our institution. Normally developed girls with no cardiac complaints who were referred for MRI for non-cardiac indications were included. Written informed consent was obtained from their guardians.

### Clinical assessment

Anthropometric measurements, including weight, height, body surface area (BSA), body mass index (BMI), BMI Z-scores and waist circumference were obtained. BMI for girls with TS was corrected for patients’ height and age to adjust for the effect of short stature^11^. Clinical data were collected including total body fat mass (kg), trunk fat mass (kg),, total cholesterol (mg/dL), triglycerides (mg/dL), HDL (mg/dL) and serum chemerin (ng/mL). Homeostasis model assessment of insulin resistance (HOMA-IR) was calculated as fasting blood glucose (mg/dL) × fasting insulin (mIU/L)/405^1^.

### Cardiac MRI protocol

Cardiac MRI studies were performed on 1.5-T MRI machine (Ingenia, Philips Healthcare, Best, The Netherlands). Free breathing retrospective ECG-gated cine steady-state free precession (SSFP) sequence was acquired. Routine short axis, 3- and 4-chambers and axial planes were obtained using the following parameters; TR = 3.2 ms, TE = 1.6 ms, field of view 256 × 256 mm to 360 × 325 mm, slice thickness = 5 mm, number of averages 1–2; and no slice gap. No contrast administration or sedation was applied.

### Cardiac MRI post processing

#### LV volume and function

Images were transferred to an offline workstation (extended MR Workspace 2.6.3.5, Philips medical systems Netherland). Semi-automated volumetric and functional analysis was performed by one experienced radiologist (7-year experience in cardiovascular imaging). The LV end diastolic (EDV) and end systolic volumes (ESV), stroke volume (SV), ejection fraction (EF) And myocardial end diastolic wall mass (LVM) were obtained. The LV mass to volume index was calculated as LVM/ LV EDV. Epicardial fat thickness and peri-hepatic fat thickness were measured^1^.

#### Cardiac MRI tissue tracking (cardiac MRI-TT)

**Fig. 1:**
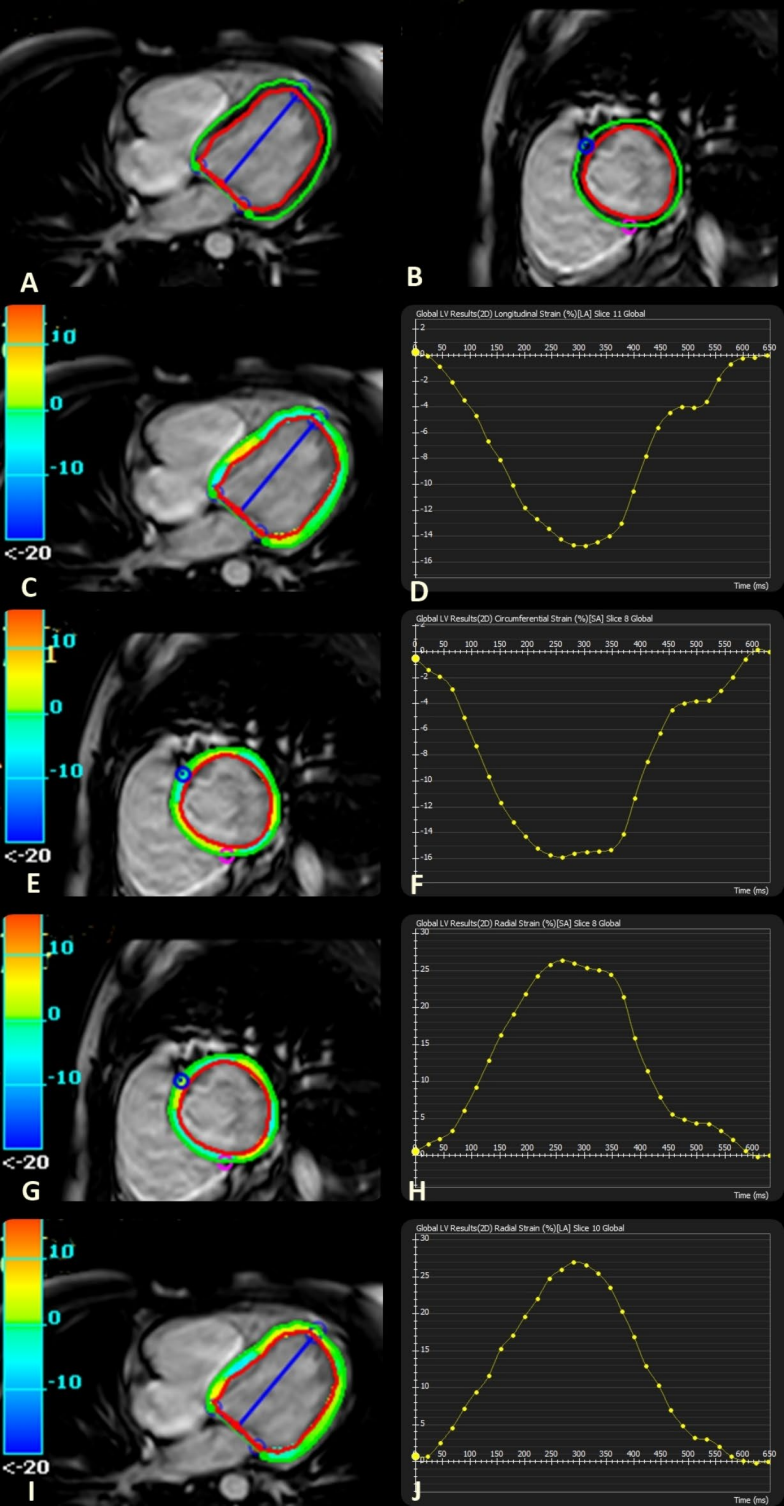
Cardiac MRI tissue tracking strain analysis in a girl with Turner syndrome. (**A**, **B**) Long- and short-axes cine SSFP images with tracings of endo- and epicardial left ventricular contours. (**C**, **D**) Color map and strain-time curve of global longitudinal strain (GLS). (**E**, **F**) Color map and strain-time curve of global circumferential strain (GCS). (**G**, **H**) Color map and strain-time curve of global radial strain short axis (GRS SAX). (**I**, **J**) Color map and strain-time curve of global radial strain long axis (GRS LAX).

Cardiac analysis software (CVi42, version 5.11, Circle Cardiovascular Imaging, Calgary, AB, Canada. https://www.circlecvi.com/cvi42) was used for measuring global LV myocardial strain parameters by an experienced radiologist (7 years of experience), blinded to the patient’s other clinical data. The LV endocardial and epicardial contours were automatically traced at end diastole in the short axis, 3- and 4-chamber and axial planes. Manual adjustment was done when needed. Global longitudinal, radial (in short and long axes) and circumferential strains were measured (GLS, GRS SAX, GRS LAX and GCS). Color coded deformation maps and strain-time curves were obtained (Fig. 1). Strain analysis was repeated for 20 patients for assessment of inter-observer agreement by a second reader (2 years of experience in cardiac imaging), blinded to the first reader’s results.

#### Aortic size, distensibility and strain

**Fig. 2:**
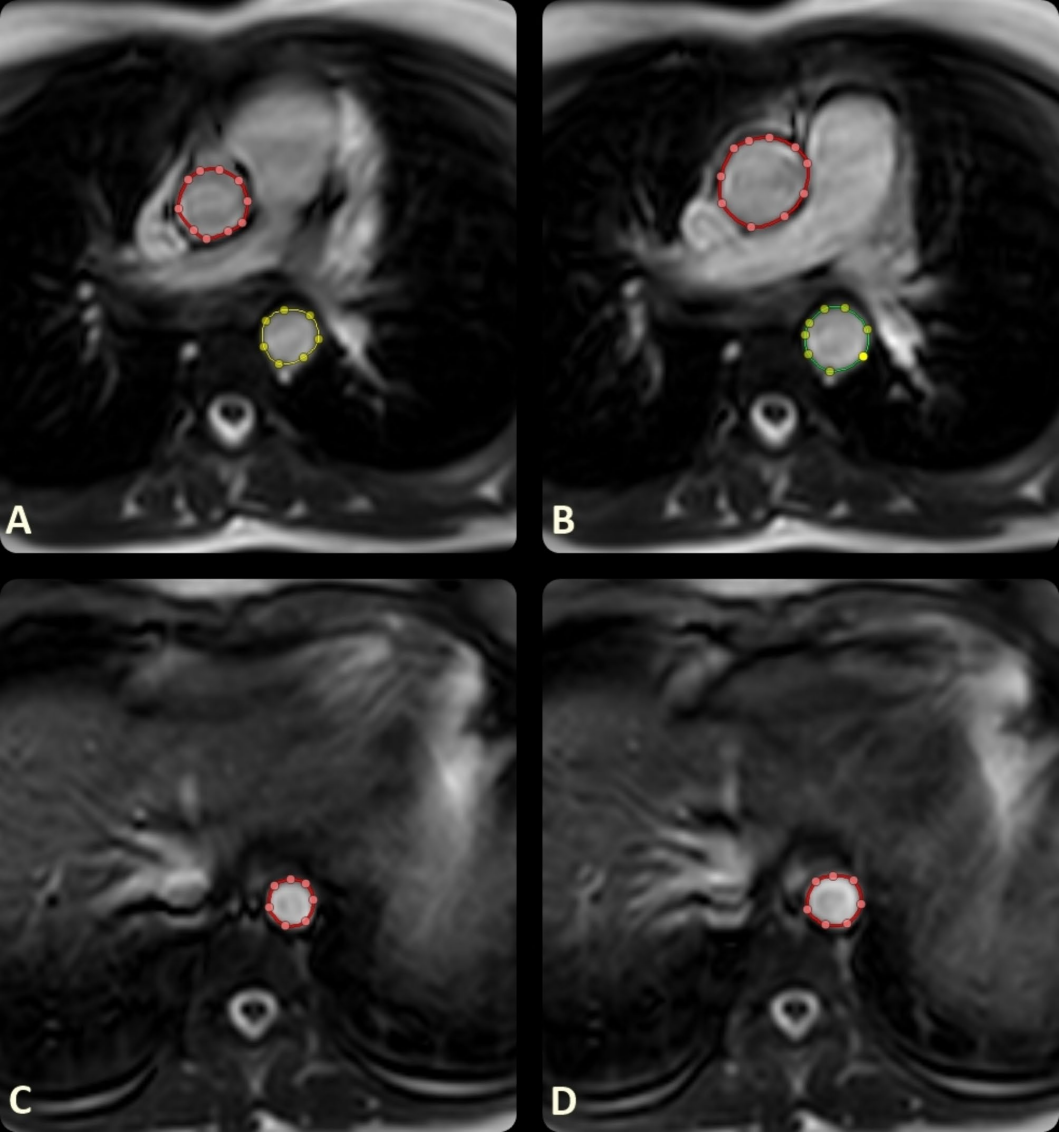
Cardiac MRI axial cine SSFP images show measurements of minimal and maximal cross sectional areas of ascending aorta and proximal descending aorta (**A**, **B**); and aorta at diaphragm (**C**, **D**).

The aorta was manually traced on axial cine SSFP images by an experienced radiologist (7 years of experience) at the end systole and end diastole to obtain the minimal and maximal aortic areas, respectively. Measurements were obtained at 3 segments: the ascending aorta, proximal descending aorta, and aorta at diaphragm (Fig. 2). Ascending aorta diameter was obtained on axial images at the end diastole.

The ascending aorta Turner-specific z-score was calculated by online calculator based on previously published data^12^. Aorta distensibility was calculated as; D = Amax-Amin / Amin * (Pmax‐Pmin). Amax and Amin are maximal and minimal aortic areas, Pmax and Pmin are the peak systolic and diastolic blood pressures and Pmin is minimal (diastolic) blood pressure. It was expressed as 1/1000 mmHg. Aortic strain (%) was calculated as; Amax – Amin /Amin * 100. Blood pressure measurements were obtained in the MRI suite immediately prior to the MRI examination.

Another experienced blinded reader (3 years of experience in cardiac imaging) repeated the aortic tracings for 20 patients to assess the inter-observer agreement.

#### Statistical analysis

Data analysis was performed using IBM SPSS Version 22.0. Normality of data was tested by Kolmogorov-Smirnov test. Data was presented as mean ± standard deviation or median and range. Differences between patients and control were assessed by Student t-test or Mann Whitney test. Correlations between myocardial strain, aortic strain and distensibility and other cardiac MRI and clinical parameters were tested by Pearson’s correlation. Interobserver agreement for myocardial strain and aortic strain and distensibility was assessed by intraclass correlation coefficients (ICCs) at 95% confidence intervals (CIs).

## Results

### Population characteristics

The study included 45 girls with TS, mean age 13.1 ± 3.2 years, and 14 control girls with mean age 11.7 ± 2.7 years, with no significant difference. Clinical, body composition and biochemical parameters among TS patients and control group are listed in Table 1.

**Table 1:** Clinical, body composition and biochemical parameters among Turner syndrome patients and control group.

	Patients (*n* = 45) Mean ± SD	Control (*n* = 14) Mean ± SD	*P* value
Age (years)	13.1 ± 3.2	11.7 ± 2.7	0.208
Weight (kg)	35.8 ± 14	38 ± 8.5	0.652
Height (cm)	131.6 ± 12	142 ± 6.6	0.01*
BSA (m^2^)	1.13 ± 0.26	1.2 ± 0.14	0.19
BMI (kg/m^2^)	19.93 ± 5.07	18.69 ± 3.87	0.47
BMI Z-score	0.92 ± 1.38ǂ	0.35 ± 1	0.234
Waist circumference (cm)	70.4 ± 10	-	-
Total body fat mass (kg)	8.5 ± 5.8	-	-
Trunk fat mass (kg)	3.4 ± 2.8	-	-
Systolic blood pressure (mmHg)	110.2 ± 10.3	118.4 ± 5.1	0.019*
Diastolic blood pressure (mmHg)	73.3 ± 8.2	78.3 ± 2.1	0.064
HOMA-IR	3.3 ± 2.7	-	-
Total cholesterol (mg/dL)	168 ± 41	-	-
Triglycerides (mg/dL)	89 ± 27	-	-
HDL (mg/dL)	68 ± 24	-	-
Chemerin (ng/mL)	272 ± 179	-	-
Epicardial fat thickness (mm)	7.9 ± 2.9	-	-
Perihepatic fat thickness (mm)	6.7 ± 3.4	-	-

*Significant P value < 0.05.

^ǂ^Height and age corrected BMI Z-score.

BSA: body surface area, BMI: body mass index, HOMA-IR: homeostasis model assessment of insulin resistance, HDL: high density lipoprotein.

TS patients were significantly shorter than controls and had higher BSA. No significant difference was found between TS patients and control in weight and BMI. The systolic blood pressure was lower in TS patients compared to the control group, but there was no significant difference in diastolic blood pressure between both groups.

Cardiac parameters are listed in Table 2. Mean LV EDV, ESV and SV were smaller in TS patients than controls. LV EF and LVM were not significantly different between the 2 groups. The LV mass to volume index was significantly increased in TS patients.

**Table 2:** Cardiac MRI parameters of left ventricle and aortic regions in Turner syndrome patients and control group.

	Patients (*n* = 45) Mean ± SD	Control (*n* = 14) Mean ± SD	*P* value
*Left ventricle*			
EF (%)	66.9 ± 6.9	67 ± 3.1	0.945
EDV (ml)	68.3 ± 22.5	91.6 ± 17.1	0.003*
ESV (ml)	22.7 ± 9.1	30.6 ± 7	0.013*
SV (ml)	45.8 ± 15.3	60.9 ± 11.3	0.005*
M (g)	40.8 ± 15	41.5 ± 10.8	0.887
LV Mass to volume index (g/ml)	0.6 ± 0.15	0.45 ± 0.09	0.004*
GLS (%)	-15.6 ± 1.8	-17.2 ± 1	0.013*
GCS (%)	-17.4 ± 2.3	-17.6 ± 2.3	0.842
GRS (SAX) (%)	29.9 ± 5.9	30.2 ± 5.9	0.869
GRS (LAX) (%)	29.3 ± 4.1	29.8 ± 6	0.756
*Ascending aorta*			
Diameter (mm)	19.5 ± 2.9	18 ± 1.6	0.236
Turner specific Z-score	-0.47 (-2.8 : 2.01)	-	-
Maximal area (mm^2^)	387 ± 122.7	426.4 ± 113.5	0.198
Minimal area (mm^2^)	296.4 ± 103.5	282.2 ± 101.2	0.326
Absolute difference (mm^2^)	90.7 ± 54.7	144.3 ± 30.1	0.002*
Strain (%)	33 ± 19	55 ± 17	0.004*
Distensibility (×10^− 3^ mm Hg^− 1^)	9.1 ± 5.5	13.9 ± 4.9	0.013*
*Proximal Descending Aorta*			
Maximal area (mm^2^)	173.7 ± 59	184.5 ± 63	0.609
Minimal area (mm^2^)	136.8 ± 56	143.4 ± 58	0.745
Absolute difference (mm^2^)	36.9 ± 25	41.4 ± 15	0.507
Strain (%)	31 ± 21	31 ± 16	0.906
Distensibility (×10^− 3^ mm Hg^− 1^)	8.4 ± 5.7	7.9 ± 4.3	0.822
*Aorta at Diaphragm*			
Maximal area (mm^2^)	146.4 ± 39	168.2 ± 52	0.137
Minimal area (mm^2^)	112.8 ± 30	125.1 ± 44	0.289
Absolute difference (mm^2^)	33.6 ± 18	43.1 ± 17	0.126
Strain (%)	31 ± 17	36 ± 14	0.371
Distensibility (×10^− 3^ mm Hg^− 1^)	8.6 ± 4.8	9.2 ± 3.6	0.726

*Significant P value < 0.05.

EF: ejection fraction, EDV: end diastolic volume, EDVi: end diastolic volume indexed, ESV: end systolic volume, ESVi: end systolic volume indexed, SV: stroke volume, SVi: stroke volume indexed, M: mass, Mi: mass indexed, GLS: global longitudinal strain, GCS: global circumferential strain, GRS (SAX) (LAX): global radial strain short axis and long axis.

All control subjects had tricuspid aortic valves; 11 out of 45 TS patients had bicuspid aortic valve (BAV). None of the patients had aortic coarctation or other aortic arch anomalies.

### Comparison of myocardial strain in TS patients and in controls

**Fig. 3:**
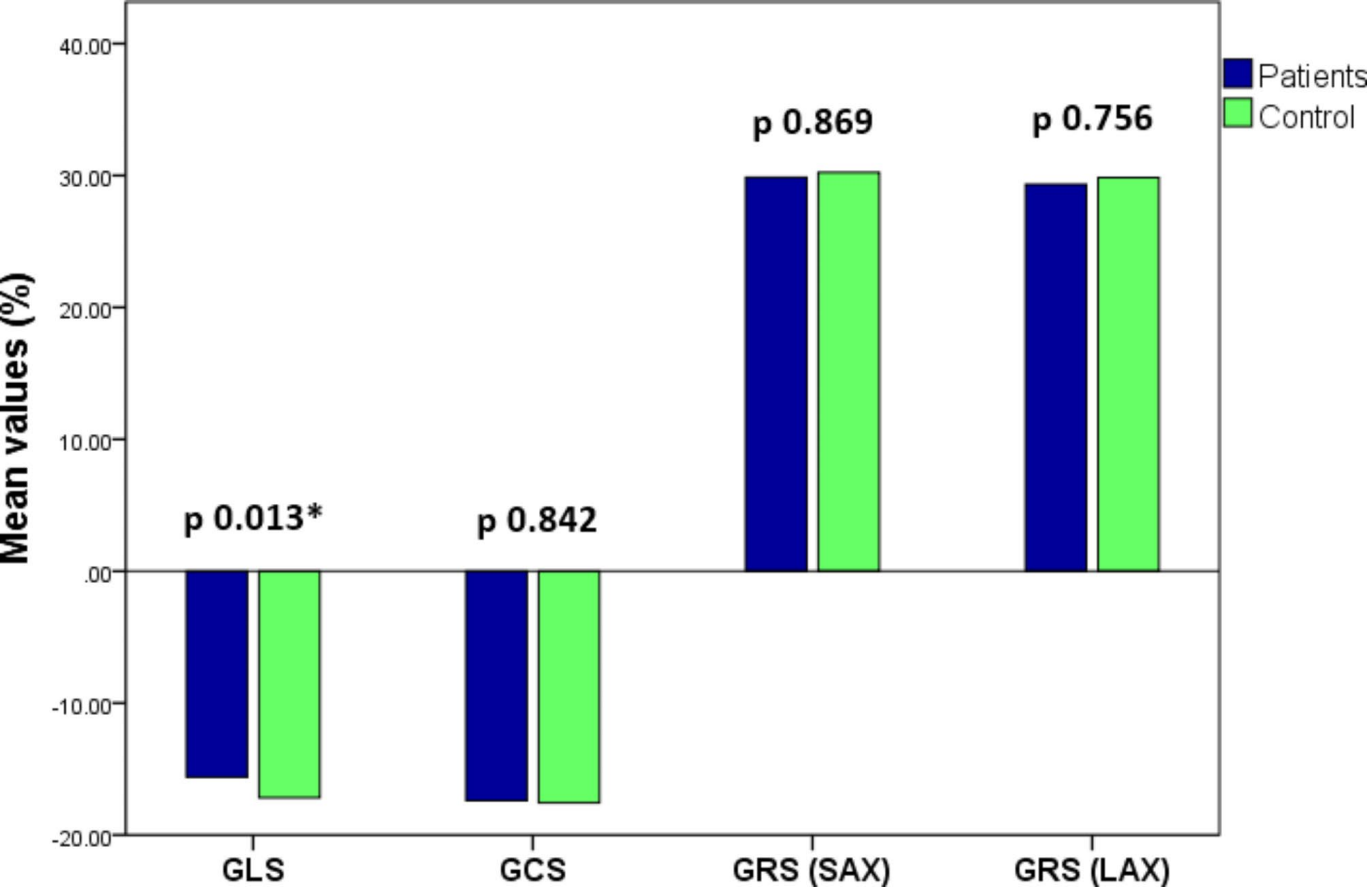
Bar charts of left ventricular myocardial strain in Turner syndrome patients versus control group. Note the significant difference in global longitudinal strain (GLS). No significant differences between patients and control in global circumferential strain (GCS), global radial strain short axis (GRS SAX) and long axis (GRS LAX).

The mean left ventricular myocardial global strain parameters are listed in Table 2 with comparison between the TS patients and control groups. The mean GLS was significantly impaired in TS patients − 15.6 ± 1.8% compared to the control group − 17.2 ± 1%, p value = 0.013. There were no significant differences in GCS, GRS LAX and GRS SAX between the 2 groups (Fig. 3).

No significant differences were observed between TS patients with TAV versus BAV in all strain parameters, Table 3.

**Table 3:** Cardiac MRI parameters of left ventricle and aortic regions between Turner syndrome patients with tricuspid aortic valve (TAV) versus bicuspid aortic valve (BAV).

	Patients with TAV(*n* = 34) Mean ± SD	Patients with BAV (*n* = 11) Mean ± SD	*P* value
*Left ventricle*			
GLS	-15.7 ± 1.8	-15.3 ± 1.7	0.525
GCS	-17.7 ± 2.5	-16.5 ± 1.6	0.130
GRS (SAX)	30.6 ± 6.2	27.4 ± 4	0.117
GRS (LAX)	30 ± 4	27.3 ± 3.4	0.059
*Ascending aorta*			
Diameter (mm)	19.7 ± 3	18.9 ± 2.8	0.486
Aortic size index (ASI) (mm/m^2^)	10.4 ± 1.2	9.7 ± 2.8	0.367
Turner specific Z-score	-0.25 ± 0.88	-0.78 ± 1.2	0.121
Maximal area (mm^2^)	383.3 ± 127	400.2 ± 114	0.499
Minimal area (mm^2^)	290.4 ± 110	315 ± 82	0.696
Absolute difference (mm^2^)	93 ± 56	85 ± 54	0.688
Strain (%)	35 ± 20	27 ± 15	0.257
Distensibility (×10^− 3^ mm Hg^− 1^)	9.6 ± 5.7	7.4 ± 4.8	0.267
*Proximal descending aorta*			
Maximal area (mm^2^)	173 ± 62.4	176 ± 51.6	0.882
Minimal area (mm^2^)	136.7 ± 57.6	137 ± 51	0.99
Absolute difference (mm^2^)	36.3 ± 28.5	39.1 ± 12.2	0.749
Strain (%)	29 ± 22	32 ± 15	0.708
Distensibility (×10^− 3^ mm Hg^− 1^)	8.2 ± 6	8.9 ± 4.8	0.744
*Aorta at diaphragm*			
Maximal area (mm^2^)	145.5 ± 35.5	149 ± 50	0.79
Minimal area (mm^2^)	112.1 ± 30.2	114.6 ± 31.4	0.812
Absolute difference (mm^2^)	33.3. ± 16	34.4 ± 22.8	0.857
Strain (%)	32 ± 17	28.7 ± 16	0.572
Distensibility (×10^− 3^ mm Hg^− 1^)	8.9 ± 4.9	7.6 ± 4.4	0.462

*Significant P value < 0.05.

TAV: tricuspid aortic valve, BAV: bicuspid aortic valve, EF: ejection fraction, EDV: end diastolic volume, EDVi: end diastolic volume indexed, ESV: end systolic volume, ESVi: end systolic volume indexed, SV: stroke volume, SVi: stroke volume indexed, M: mass, Mi: mass indexed, GLS: global longitudinal strain, GCS: global circumferential strain, GRS (SAX) (LAX): global radial strain short axis and long axis.

Among clinical parameters, the GLS in TS patients was significantly correlated to age *r*=-0.493, p 0.001; weight *r*= -0.310, *p* = 0.038; height *r*= -0.449, *p* = 0.002 and diastolic BP *r*=-0.313, *p* = 0.033. Among other left ventricular cardiac MRI parameters, GLS was correlated to EDV *r*= -0.368, *p* = 0.014; ESV *r*=-0.328, *p* = 0.03; and SV *r*=-0.337, *p* = 0.025, and LV mass to volume index *r* = 0.349, *p* = 0.01. Correlations are listed in supplementary material. Multi-variate linear regression model did not identify any independent risk factor for myocardial strain impairment.

### Comparison of aortic measurements in TS patients and in control group

The mean aortic diameters, minimal and maximal areas, aortic strain and distensibility in the ascending aorta, proximal descending aorta and the aorta at diaphragm for TS patients and control groups are listed in Table 2. There was no significant difference in aortic diameter between the patients and control groups.

**Fig. 4:**
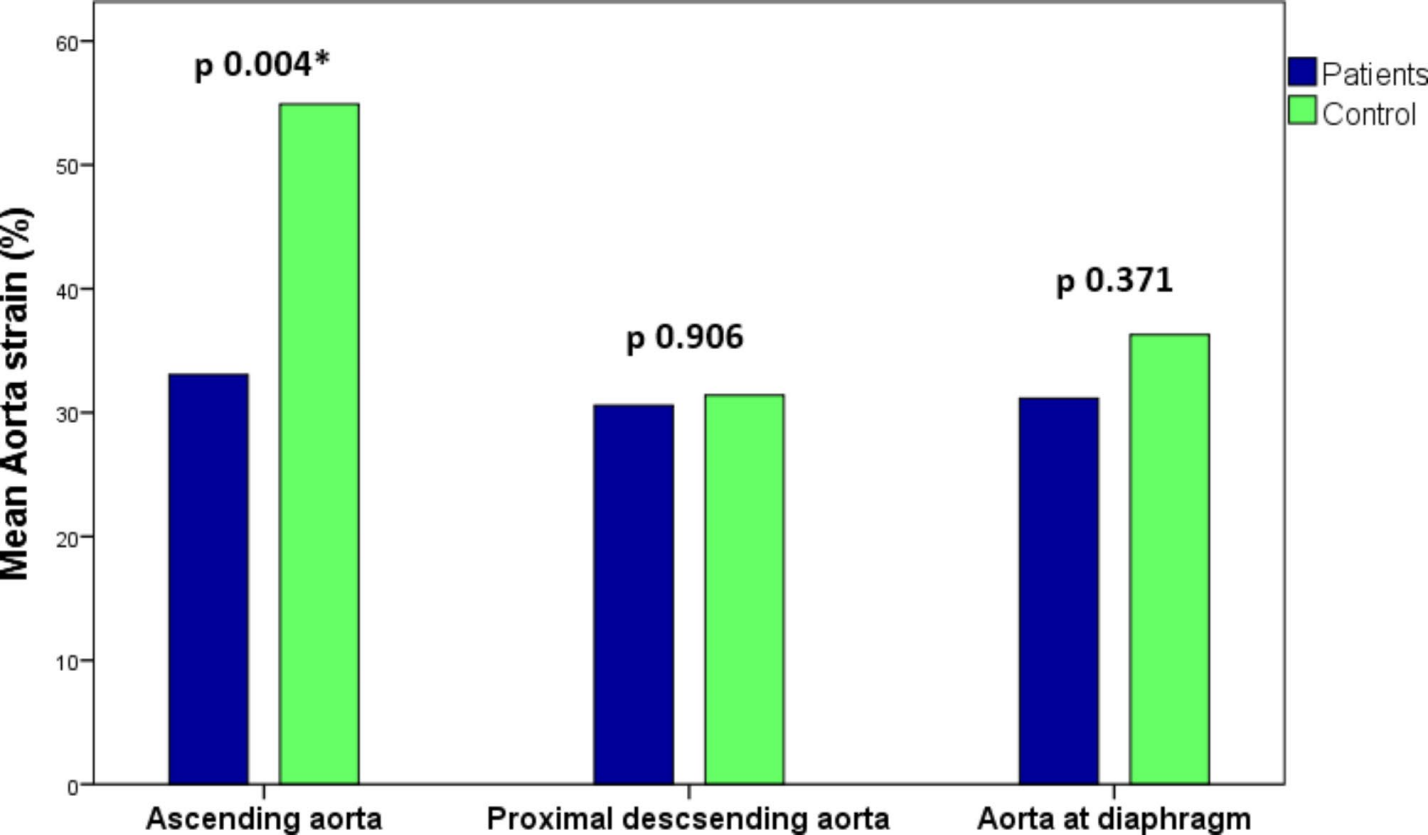
Bar charts of aortic strain in Turner syndrome patients versus control group. Note the significant difference in ascending aorta strain between patients and control, and the non significant differences in the proximal descending aorta and aorta at diaphragm.

**Fig. 5:**
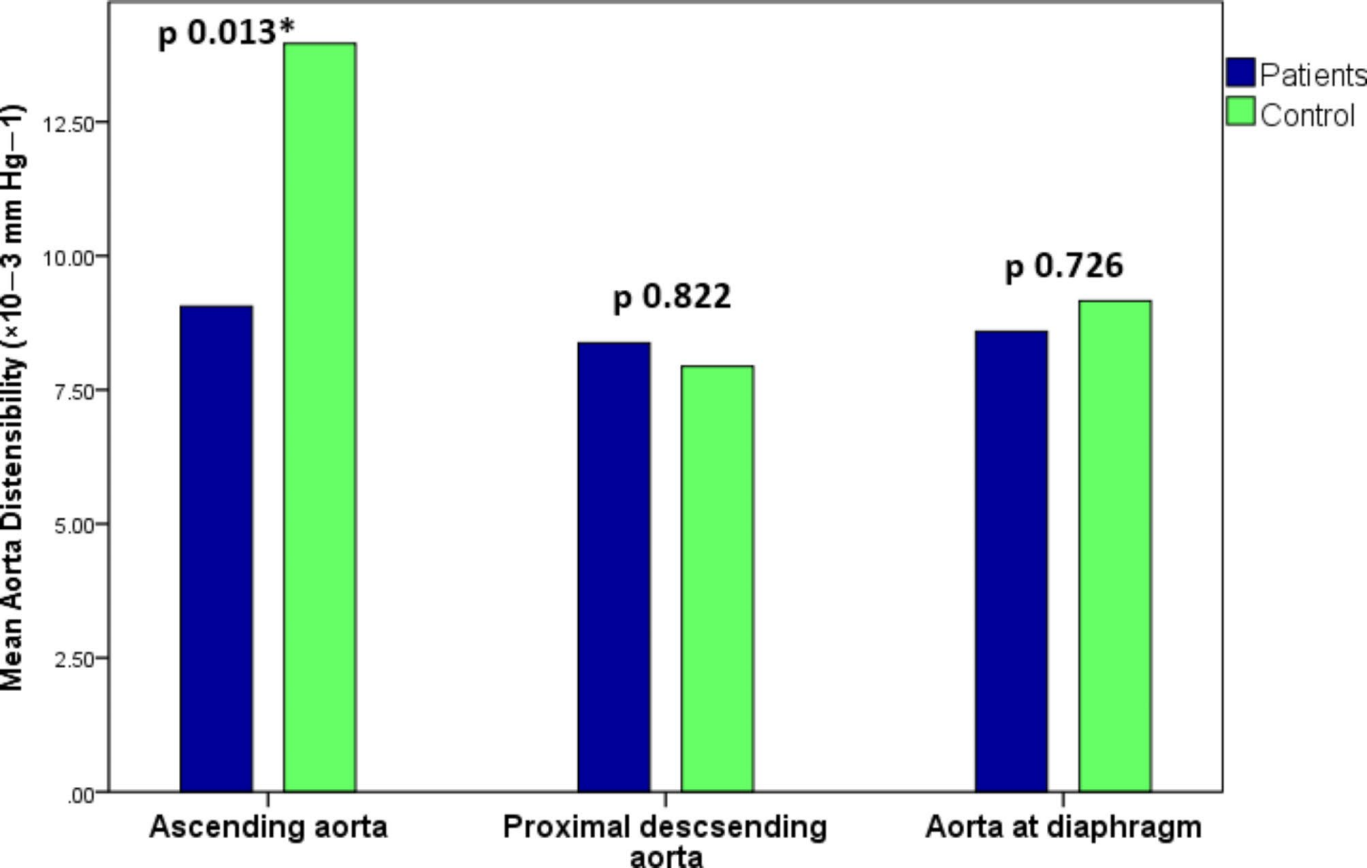
Bar charts of aortic distensibility in Turner syndrome patients versus control group. Note the significant difference in ascending aorta distensibility between patients and control, and the non significant differences in the proximal descending aorta and aorta at diaphragm.

The mean ascending aorta strain was significantly lower in TS patients 33 ± 19% compared to the control group 55 ± 17%, p value = 0.004. The mean ascending aorta distensibility was significantly lower in TS patients 9.1 ± 5.5 compared to the control group 13.9 ± 4.9 10^− 3^ mm Hg^− 1^, p value = 0.013. There were no significant differences in aortic strain and distensibility of the proximal descending aorta and aorta at diaphragm between the 2 groups (Figs. 4 and 5).

No significant differences were observed between TS patients with TAV versus BAV in aortic strain and distensibility in the 3 aortic regions, Table 3.

The ascending aorta strain was correlated to LV EF *r*= -0.333, *p* = 0.027. No significant correlations were found between LV myocardial global strain parameters and aortic strain and distensibility. Correlations are listed in supplementary material. Multi-variate linear regression model did not identify any independent risk factor for reduced aortic strain and distensibility.

### Inter-observer agreement for myocardial strain and aortic distensibility measurements

Analyses revealed substantial inter-observer agreement for myocardial GLS (ICC = 0.61, 95% CI -0.08: 0.86), GCS (ICC = 0.70, 95% CI 0.14: 0.87), and GRS LAX (ICC = 0.64, 95% CI 0.02: 0.87), and almost perfect agreement for GRS SAX (ICC = 0.82, 95% CI 0.53: 0.93). There was almost perfect agreement for ascending aorta distensibility and strain measurements (ICC = 0.95, 95% CI 0.87 :0.98), proximal descending aorta distensibility (ICC = 0.94, 95% CI 0.85 :0.976) and strain (ICC = 0.94, CI 0.84: 0.98), and aorta at diaphragm distensibility (ICC = 0.93, 95% CI 0.83: 0.98) and strain (ICC = 0.91, 95% CI: 0.78: 0.97).

## Discussion

The results of this cardiac MRI study demonstrated impaired LV myocardial GLS in young TS patients with no cardiac complaints and preserved LV EF, compared to healthy control group. Regional assessment of the thoracic aorta revealed reduced ascending aorta strain and distensibility in TS patients compared to control, with no difference in aortic size.

### LV myocardial strain

To our knowledge, this is the first study to report LV strain analysis by cardiac MRI tissue tracking in TS patients. Results of previous echocardiography studies were inconsistent^13^. Some studies found no significant differences in myocardial strain between TS patients and control subjects^6,13^. Other previous echocardiography studies, in line with our cardiac MRI results, reported impaired LV myocardial strain in TS patients^2,7,14–16^.

In our study, only GLS was impaired, while GCS and GRS were not. GLS is mainly concerned with longitudinal myocardial fibers, predominant in the subendocardial layer^17^. GCS reflects the circumferentially oriented muscle fibers in the mid wall. GRS is concerned with myocardial deformation towards the center of the LV cavity^17^. The earlier involvement of GLS might be explained by the assumed vulnerability of subendocardial longitudinal myocardial fibers to cardiovascular risk factors common in TS patients such as such as diabetes, insulin resistance hypertension or overweight^2,14^. Another proposed explanation in the literature for reduced GLS in TS patients is the common association between TS and presence of aortic coarctation or BAV. Aortic coarctation causes pressure overload on the LV leading to increased myocardial wall stress and systolic dysfunction. BAV causes altered aortic hemodynamics with secondary increase in LV after load and myocardial remodeling^2,16^. Our cohort did not include patients with aortic coarctation, and no significant difference in myocardial strain was observed between TS patients with BAV versus TAV suggesting that myocardial involvement in our study is not secondary to the extrinsic effect of aortic morphology. A third explanation for reduced GLS in TS patients is the reduced aortic distensibility with subsequent increased LV afterload leading to impaired myocardial strain. In addition to the aforementioned extrinsic factors, it is still reasonable to assume that impaired myocardial deformation in TS is also caused by an intrinsic myocardial component^7^.

### Aortic distensibility

Aortic size is the main parameter used to assess aortic dissection risk in TS patients^3^. In the current study, the aorta was not dilated in TS patients compared to control. However, we demonstrated impaired strain and distensibility of the ascending aorta despite the absence of dilatation.

These results highlight the importance of assessment of functional elasticity of the aorta in addition to size, especially in younger age^18^.

Functional assessment of the aorta may be obtained by different methodologies, but MRI offers the advantage of segmental measurements at the different aortic levels. In the current study and in line with a previous study, impaired elasticity was detected in the ascending aorta only suggesting preferential involvement of proximal aorta in TS patients^4^.

The ascending aorta was stiffer in TS patients with BAV compared to those with TAV, but the difference did not reach statistical significance. The current results support previous reports that aortic stiffness is not caused by- or exclusive to- TS patients with BAV, suggestive of intrinsic aortic wall defect in TS patients, with likely an additional effect of BAV on the intrinsic wall abnormalities^4,19,20^. The young age of our cohort might explain the non-dilated aorta in TS patients with BAV.

### LV hypertrophy and volume

The LV volumes were significantly reduced in TS patients compared to the control group, which is in line with other previous echocardiography studies^21,22^.

The LV mass in our TS patients was comparable to that of controls. There is discrepancy in the literature concerning LV hypertrophy in TS patients, where several authors reported LV hypertrophy in TS patients by echocardiography^6,15,23,24^, while other studies did not demonstrate increased LV mass^13,14,21,22^. This discrepancy in the results of LV mass may be due to differences in measurement methods^13^, or the use of indexing to BSA^23^. In our study we did not use indexing to BSA to avoid misleading results due to short stature of TS patients, with significant difference in their BSA compared to age matched controls.

Interestingly, although the LV was smaller in our cohort of TS patients, the LV mass was comparable to the control. This mismatch between mass and volume is better expressed by using the LV mass to volume index^25,26^. We did find higher mass to volume index in TS patients compared to controls, reflecting disproportionately larger mass values for smaller LV cavity volumes.

### Study limitations

One limitation of this study is that the contributing effects of growth hormone and estrogen replacement therapies on myocardial and aortic functions could not be evaluated, because most of the patients were already receiving or had received growth hormone and estrogen replacement therapy at the time of study. Another limitation is the small number of the TS BAV subgroup, with only 11 patients. This might affect the reliability of ruling out the effect of aortic valve morphology on myocardial and aortic functional parameters. Also, the absence of aortic coarctation or other aortic arch anomalies in our patients is a limitation.

Although multiple significant correlations were observed between myocardial and aortic functional parameters with clinical, biochemical and other cardiac MRI parameters; multi-variate regression analysis did not identify any independent risk factor for myocardial strain or aortic elasticity impairment. In a previous MRI study, age was the main determinant for aortic stiffness^4^; however, their study included a wide age range (13–59 years), compared to the narrow age range of our homogeneous group of TS patients (10–16 years) which eliminated the effect of age on myocardial and aortic functional properties.

Lastly, the cross-sectional nature of the study does not allow assumptions regarding progression of the detected myocardial and aortic abnormalities with time. Further longitudinal studies are recommended.

## Conclusion

This cardiac MRI study investigated LV myocardial deformation and aortic distensibility offering important functional data in TS patients. LV myocardial GLS was impaired in TS patients with no cardiac complaints and preserved LV EF. Ascending aorta strain and distensibility were reduced in TS patients with non-dilated aorta. Cardiac MRI measurements of LV deformation and aortic function carry potential value as markers for early detection of myocardial and aortic disease in TS patients. Validation of the clinical impact and prognostic role require further longitudinal studies.

We found increased mass to volume index in TS patients, reflecting disproportionately larger mass values for smaller LV cavity volumes. We suggest the use of this index in TS patients because of their smaller LV volumes.

## Electronic supplementary material

Below is the link to the electronic supplementary material.

Supplementary Material 1

## Data Availability

Data availabilityThe datasets used and analysed during the current study are available from the corresponding author on reasonable request.

## References

[1] Salem, N. A. et al. Epicardial and perihepatic fat as cardiometabolic risk predictors in girls with turner syndrome: A cardiac magnetic resonance study. *J. Clin. Res. Pediatr. Endocrinol.* **13**, 408–417 (2021). 34013713 10.4274/jcrpe.galenos.2021.2021.0030 PMC8638625

[2] Oberhoffer, F.S., Abdul-Khaliq, H., Jung, A.M., Rohrer, T.R. & Abd El Rahman, M. Left ventricular remodelling among Turner syndrome patients: Insights from non-invasive 3D echocardiography-derived pressure-volume loop analysis. *Clin. Res. Cardiol.* **109**, 892–903 (2020). 10.1007/s00392-019-01579-8 31786629

[3] Silberbach, M., *et al*. American Heart Association Council on cardiovascular disease in the young; Council on genomic and precision medicine; and council on peripheral vascular disease. Cardiovascular Health in Turner Syndrome: A Scientific Statement From the American Heart Association. *Circ. Genom. Precis. Med.* **11**, e000048 (2018). 10.1161/HCG.0000000000000048 30354301

[4] Devos, D. G. et al. Proximal aortic stiffening in Turner patients may be present before dilation can be detected: A segmental functional MRI study. *J. Cardiovasc. Magn. Reson.* **19**, 27 (2017). 28222756 10.1186/s12968-017-0331-0 PMC5320803

[5] Wen, J. et al. Impaired aortic distensibility and elevated central blood pressure in Turner syndrome: A cardiovascular magnetic resonance study. *J. Cardiovasc. Magn. Reson.* **20**, 80 (2018). 30541571 10.1186/s12968-018-0497-0 PMC6292015

[6] Oberhoffer, F. S. et al. Assessment of left ventricular myocardial work in Turner syndrome patients: Insights from the novel non-invasive pressure-strain loop analysis method. *Quant. Imaging Med. Surg.* **10**, 15–25 (2020). 31956525 10.21037/qims.2019.09.19 PMC6960421

[7] Andersen, N. H. et al. Subclinical left ventricular dysfunction in normotensive women with Turner’s syndrome. *Heart* **92**, 1516–1517 (2006). 16973807 10.1136/hrt.2005.081471 PMC1861017

[8] Sobh, D. M. et al. Left ventricular strain analysis by tissue tracking-cardiac magnetic resonance for early detection of cardiac dysfunction in children with end-stage renal disease. *J. Magn. Reason. Imaging* **54**, 1476–1485 (2021). 10.1002/jmri.27700 34037288

[9] Tawfik, A. M. et al. Right ventricular strain analysis by tissue tracking cardiac magnetic resonance imaging in pediatric patients with end-stage renal disease. *J. Thorac. Imaging* **39**, 49–56 (2024). 37265246 10.1097/RTI.0000000000000716

[10] Sobh, D. M. et al. Impaired aortic strain and distensibility by cardiac MRI in children with chronic kidney disease. *Sci. Rep.* **12**, 11079 (2022). 35773282 10.1038/s41598-022-15017-9 PMC9247100

[11] Bonthuis, M. et al. Application of body mass index according to height-age in short and tall children. *PLoS ONE* **8**, e72068 (2013). 23951283 10.1371/journal.pone.0072068 PMC3737143

[12] Quezada, E., Lapidus, J., Shaughnessy, R., Chen, Z. & Silberbach, M. Aortic dimensions in Turner syndrome. *Am. J. Med. Genet. A* **167**, 2527–2532 (2015). 10.1002/ajmg.a.37208 26118429

[13] Noordman, I. D. et al. Cardiac abnormalities in girls with Turner syndrome: ECG abnormalities, myocardial strain imaging, and karyotype-phenotype associations. *Am. J. Med. Genet. A* **185**, 2399–2408 (2021). 33969942 10.1002/ajmg.a.62259 PMC8359841

[14] AbdelMassih, A. F., Attia, M., Ismail, M. M. & Samir, M. Insulin resistance linked to subtle myocardial dysfunction in normotensive Turner syndrome young patients without structural heart diseases. *J. Pediatr. Endocrinol. Metab.* **31**, 1355–1361 (2018). 30433872 10.1515/jpem-2018-0207

[15] Oz, F. et al. Doppler-derived strain imaging detects left ventricular systolic dysfunction in children with Turner syndrome. *Echocardiography* **31**, 1017–1022 (2014). 24410871 10.1111/echo.12500

[16] Hoven, A. T. et al. Systolic and diastolic strain measurements show left ventricular dysfunction in women with turner syndrome. *Congenital Heart Dis.* **16**, 357–368 (2021).

[17] Muser, D., Castro, S. A., Santangeli, P. & Nucifora, G. Clinical applications of feature-tracking cardiac magnetic resonance imaging. *World J. Cardiol.* **10**, 210–221 (2018). 30510638 10.4330/wjc.v10.i11.210 PMC6259029

[18] Schäfer, M. et al. Aortic stiffness in adolescent Turner and Marfan syndrome patients. *Eur. J. Cardiothorac. Surg.* **54**, 926–932 (2018). 29684119 10.1093/ejcts/ezy168

[19] De Groote, K. et al. Increased aortic stiffness in prepubertal girls with Turner syndrome. *J. Cardiol.* **69**, 201–207 (2017). 27056149 10.1016/j.jjcc.2016.03.006

[20] Pees, C. et al. Aortic elasticity deterioration proves intrinsic abnormality of the ascending aorta in pediatric Turner syndrome unrelated to the aortic valve morphology. *Heart Vessels* **33**, 1350–1357 (2018). 29777298 10.1007/s00380-018-1187-4 PMC6208677

[21] Van den Berg, J. et al. Cardiac status after childhood growth hormone treatment of Turner syndrome. *J. Clin. Endocrinol. Metab.* **93**, 2553–2558 (2008). 18430775 10.1210/jc.2007-2313

[22] Obara-Moszynska, M.,* et al.* The usefulness of magnetic resonance imaging of the cardiovascular system in the diagnostic work-up of patients with turner syndrome. *Front. Endocrinol.* **16**, 609 (2018). 10.3389/fendo.2018.00609 PMC6232706 30459711

[23] Sozen, A. B. et al. Left ventricular thickness is increased in nonhypertensive Turner’s syndrome. *Echocardiography* **26**, 943–949 (2009). 19486113 10.1111/j.1540-8175.2009.00902.x

[24] Mortensen, K. H., Gravholt, C. H., Hjerrild, B. E., Stochholm, K. & Andersen, N. H. Left ventricular hypertrophy in Turner syndrome: A prospective echocardiographic study. *Echocardiography* **29**, 1022–1030 (2012). 22758401 10.1111/j.1540-8175.2012.01754.x

[25] Scalia, G., Hamilton-Craig, C., Slaughter, R. & Cain, P. Left ventricular mass-volume index: A new parameter for the assessment of “disproportionate” left ventricular hypertrophy by echocardiography. *Heart Lung Circ* **20S**, S1–S155 (2011).

[26] Lee, S., James, N. C. & Srivastava, S. U. S. Simplifying the diagnosis of left ventricular hypertrophy: Is left ventricular mass to volume ratio constant in children throughout growth?. *J. Cardiovasc. Magn. Reason.* **18**, 122 (2016).

