# Supplementary material for: Impaired myocardial deformation and aortic distensibility by cardiac MRI in girls with Turner syndrome
Source: Sci Rep. 2025 Mar 12;15:8457. doi: 10.1038/s41598-024-75312-5 (PMC11897320; doi:10.1038/s41598-024-75312-5)
Supplement: Supplementary file 1 — Supplementary Material 1 [file 41598_2024_75312_MOESM1_ESM.docx]

**Supplementary correlations table 1**

|  | | GLS | GCS | GRSsax | GRSlax |
| --- | --- | --- | --- | --- | --- |
| age | Pearson Correlation | -.493** | -,236 | ,277 | .372* |
|  | Sig. (2-tailed) | ,001 | ,119 | ,065 | ,012 |
| weight | Pearson Correlation | -.310* | -,060 | ,101 | ,133 |
|  | Sig. (2-tailed) | ,038 | ,693 | ,509 | ,384 |
| Height | Pearson Correlation | -.449** | -,247 | ,284 | .333* |
|  | Sig. (2-tailed) | ,002 | ,101 | ,059 | ,025 |
| BSA | Pearson Correlation | -,266 | ,065 | -,023 | ,084 |
|  | Sig. (2-tailed) | ,077 | ,671 | ,880 | ,582 |
| BSAmodified | Pearson Correlation | -.376* | -,105 | ,149 | ,200 |
|  | Sig. (2-tailed) | ,011 | ,493 | ,329 | ,187 |
| BMI | Pearson Correlation | -,247 | ,056 | -,019 | ,062 |
|  | Sig. (2-tailed) | ,102 | ,713 | ,899 | ,685 |
| systBP | Pearson Correlation | -,242 | -,126 | ,149 | ,271 |
|  | Sig. (2-tailed) | ,109 | ,410 | ,329 | ,072 |
| diastBP | Pearson Correlation | -.318* | -,038 | ,052 | .313* |
|  | Sig. (2-tailed) | ,033 | ,807 | ,737 | ,036 |
| BMIzHA | Pearson Correlation | -,221 | ,077 | -,042 | -,002 |
|  | Sig. (2-tailed) | ,145 | ,615 | ,782 | ,991 |
| WC | Pearson Correlation | -,185 | ,111 | -,066 | ,015 |
|  | Sig. (2-tailed) | ,223 | ,469 | ,669 | ,923 |
| TBFATMASS | Pearson Correlation | -,096 | ,027 | ,009 | -,076 |
|  | Sig. (2-tailed) | ,533 | ,860 | ,952 | ,617 |
| TrunKfatmass | Pearson Correlation | -,053 | ,028 | -,006 | -,108 |
|  | Sig. (2-tailed) | ,729 | ,856 | ,971 | ,480 |
| HomaIR | Pearson Correlation | -,145 | ,179 | -,025 | ,107 |
|  | Sig. (2-tailed) | ,422 | ,320 | ,889 | ,554 |
| Cholesterol | Pearson Correlation | -,117 | -,164 | ,209 | ,030 |
|  | Sig. (2-tailed) | ,517 | ,360 | ,243 | ,869 |
| HDL | Pearson Correlation | -,043 | ,253 | -,182 | -,028 |
|  | Sig. (2-tailed) | ,813 | ,155 | ,311 | ,875 |
| TRIGLYCERIDES | Pearson Correlation | ,140 | ,076 | ,010 | -,194 |
|  | Sig. (2-tailed) | ,438 | ,673 | ,955 | ,280 |
| srchemerin | Pearson Correlation | -,310 | -,258 | .360* | ,268 |
|  | Sig. (2-tailed) | ,079 | ,147 | ,040 | ,132 |
| EFTdixonSA | Pearson Correlation | ,080 | -,075 | -,095 | -,005 |
|  | Sig. (2-tailed) | ,656 | ,678 | ,599 | ,979 |
| Perihepaticfat | Pearson Correlation | ,082 | -,211 | ,132 | -,076 |
|  | Sig. (2-tailed) | ,651 | ,238 | ,465 | ,673 |
| Ascaortadiameter | Pearson Correlation | -,215 | -,137 | ,167 | ,012 |
|  | Sig. (2-tailed) | ,157 | ,368 | ,272 | ,940 |
| Tzscore | Pearson Correlation | .321* | -,099 | ,117 | -,162 |
|  | Sig. (2-tailed) | ,031 | ,519 | ,443 | ,289 |
| Mtovolumeindex | Pearson Correlation | ,288 | ,029 | ,022 | -.336* |
|  | Sig. (2-tailed) | ,058 | ,851 | ,885 | ,026 |
| asi | Pearson Correlation | ,181 | -,098 | ,074 | -,132 |
|  | Sig. (2-tailed) | ,233 | ,523 | ,631 | ,386 |

**Supplementary correlations Table 2**

|  | | AscStrain | AscDist | PrxDAstrain | PrxDADist | AodiaphStrain | AodiaphDist |
| --- | --- | --- | --- | --- | --- | --- | --- |
| age | Pearson Correlation | -,156 | -,230 | -,120 | -,212 | -,034 | -,119 |
|  | Sig. (2-tailed) | ,305 | ,129 | ,431 | ,162 | ,824 | ,435 |
| weight | Pearson Correlation | -,108 | -,117 | -,201 | -,212 | -,099 | -,108 |
|  | Sig. (2-tailed) | ,482 | ,445 | ,186 | ,161 | ,518 | ,482 |
| Height | Pearson Correlation | -,149 | -,219 | -,284 | -.360^*^ | -,046 | -,128 |
|  | Sig. (2-tailed) | ,328 | ,148 | ,059 | ,015 | ,764 | ,403 |
| BSA | Pearson Correlation | -,058 | -,021 | -,138 | -,109 | -,113 | -,068 |
|  | Sig. (2-tailed) | ,707 | ,892 | ,366 | ,474 | ,459 | ,658 |
| BSAmodified | Pearson Correlation | -,119 | -,141 | -,225 | -,253 | -,087 | -,111 |
|  | Sig. (2-tailed) | ,436 | ,354 | ,136 | ,094 | ,570 | ,467 |
| BMI | Pearson Correlation | -,059 | -,028 | -,135 | -,111 | -,104 | -,067 |
|  | Sig. (2-tailed) | ,700 | ,855 | ,375 | ,467 | ,498 | ,662 |
| systBP | Pearson Correlation | -,132 | -,244 | -,020 | -,142 | -,057 | -,217 |
|  | Sig. (2-tailed) | ,389 | ,107 | ,897 | ,352 | ,712 | ,153 |
| diastBP | Pearson Correlation | -,203 | -,205 | -,081 | -,106 | -,137 | -,137 |
|  | Sig. (2-tailed) | ,181 | ,177 | ,597 | ,490 | ,370 | ,371 |
| BMIzHA | Pearson Correlation | -,037 | -,002 | -,136 | -,126 | -,037 | ,027 |
|  | Sig. (2-tailed) | ,810 | ,992 | ,372 | ,411 | ,807 | ,862 |
| WC | Pearson Correlation | -,102 | -,085 | -,065 | -,065 | -,022 | ,004 |
|  | Sig. (2-tailed) | ,506 | ,580 | ,673 | ,671 | ,888 | ,981 |
| TBFATMASS | Pearson Correlation | -,107 | -,095 | -,250 | -,232 | -,046 | -,043 |
|  | Sig. (2-tailed) | ,483 | ,534 | ,097 | ,126 | ,762 | ,778 |
| TrunKfatmass | Pearson Correlation | -,138 | -,126 | -,215 | -,195 | -,039 | -,059 |
|  | Sig. (2-tailed) | ,365 | ,411 | ,155 | ,199 | ,798 | ,702 |
| HomaIR | Pearson Correlation | -,028 | -,067 | -,125 | -,118 | -,092 | -,123 |
|  | Sig. (2-tailed) | ,879 | ,710 | ,490 | ,512 | ,611 | ,495 |
| Cholesterol | Pearson Correlation | -,063 | -,008 | -,053 | -,033 | ,316 | .370^*^ |
|  | Sig. (2-tailed) | ,727 | ,966 | ,770 | ,856 | ,073 | ,034 |
| HDL | Pearson Correlation | -,135 | -,113 | -,075 | -,024 | ,223 | ,284 |
|  | Sig. (2-tailed) | ,453 | ,531 | ,679 | ,897 | ,213 | ,109 |
| TRIGLYCERIDES | Pearson Correlation | -,068 | -,125 | -,140 | -,149 | -,202 | -,222 |
|  | Sig. (2-tailed) | ,705 | ,488 | ,436 | ,408 | ,260 | ,214 |
| srchemerin | Pearson Correlation | -,099 | -,107 | ,182 | ,163 | ,048 | ,026 |
|  | Sig. (2-tailed) | ,585 | ,555 | ,311 | ,366 | ,792 | ,885 |
| EFTdixonSA | Pearson Correlation | -,108 | -,074 | -.363^*^ | -,320 | -,010 | ,005 |
|  | Sig. (2-tailed) | ,551 | ,683 | ,038 | ,065 | ,958 | ,979 |
| Perihepaticfat | Pearson Correlation | ,015 | -,012 | -,299 | -,303 | -.396^*^ | -.405^*^ |
|  | Sig. (2-tailed) | ,935 | ,948 | ,091 | ,086 | ,023 | ,020 |
| Ascaortadiameter | Pearson Correlation | -,180 | -,175 | -,146 | -,118 | -,009 | ,060 |
|  | Sig. (2-tailed) | ,235 | ,251 | ,338 | ,439 | ,954 | ,694 |
| Tzscore | Pearson Correlation | -,192 | -,182 | ,029 | ,050 | ,285 | ,293 |
|  | Sig. (2-tailed) | ,205 | ,233 | ,851 | ,745 | ,058 | ,051 |
| Mtovolumeindex | Pearson Correlation | -,193 | -,171 | -,004 | ,016 | ,196 | ,186 |
|  | Sig. (2-tailed) | ,210 | ,268 | ,979 | ,918 | ,203 | ,227 |
| asi | Pearson Correlation | -,057 | -,093 | ,043 | ,035 | ,037 | ,030 |
|  | Sig. (2-tailed) | ,711 | ,546 | ,782 | ,819 | ,810 | ,844 |

**Supplementary table 3**

|  | | GLS | GCS | GRSsax | GRSlax | AscStrain | AscDist | PrxDAstrain | PrxDADist | AodiaphStrain | AodiaphDist |
| --- | --- | --- | --- | --- | --- | --- | --- | --- | --- | --- | --- |
| GLS | Pearson Correlation | 1 | ,152 | -,231 | -.724^**^ | -,059 | -,052 | -,029 | ,005 | ,035 | ,043 |
|  | Sig. (2-tailed) |  | ,319 | ,127 | ,000 | ,698 | ,734 | ,851 | ,973 | ,822 | ,781 |
| GCS | Pearson Correlation | ,152 | 1 | -.914^**^ | -,188 | ,071 | ,103 | ,128 | ,185 | ,001 | ,042 |
|  | Sig. (2-tailed) | ,319 |  | ,000 | ,217 | ,643 | ,502 | ,402 | ,225 | ,992 | ,785 |
| GRSsax | Pearson Correlation | -,231 | -.914^**^ | 1 | ,205 | -,082 | -,114 | -,119 | -,180 | ,084 | ,032 |
|  | Sig. (2-tailed) | ,127 | ,000 |  | ,177 | ,593 | ,455 | ,436 | ,236 | ,584 | ,835 |
| GRSlax | Pearson Correlation | -.724^**^ | -,188 | ,205 | 1 | -,031 | -,029 | ,047 | -,006 | ,077 | ,039 |
|  | Sig. (2-tailed) | ,000 | ,217 | ,177 |  | ,838 | ,849 | ,757 | ,969 | ,613 | ,799 |
| AscStrain | Pearson Correlation | -,059 | ,071 | -,082 | -,031 | 1 | .966^**^ | ,286 | ,273 | ,179 | ,188 |
|  | Sig. (2-tailed) | ,698 | ,643 | ,593 | ,838 |  | ,000 | ,057 | ,069 | ,240 | ,215 |
| AscDist | Pearson Correlation | -,052 | ,103 | -,114 | -,029 | .966^**^ | 1 | ,254 | ,290 | ,205 | ,282 |
|  | Sig. (2-tailed) | ,734 | ,502 | ,455 | ,849 | ,000 |  | ,092 | ,054 | ,176 | ,060 |
| PrxDAstrain | Pearson Correlation | -,029 | ,128 | -,119 | ,047 | ,286 | ,254 | 1 | .971^**^ | ,117 | ,091 |
|  | Sig. (2-tailed) | ,851 | ,402 | ,436 | ,757 | ,057 | ,092 |  | ,000 | ,444 | ,551 |
| PrxDADist | Pearson Correlation | ,005 | ,185 | -,180 | -,006 | ,273 | ,290 | .971^**^ | 1 | ,109 | ,144 |
|  | Sig. (2-tailed) | ,973 | ,225 | ,236 | ,969 | ,069 | ,054 | ,000 |  | ,477 | ,345 |
| AodiaphStrain | Pearson Correlation | ,035 | ,001 | ,084 | ,077 | ,179 | ,205 | ,117 | ,109 | 1 | .944^**^ |
|  | Sig. (2-tailed) | ,822 | ,992 | ,584 | ,613 | ,240 | ,176 | ,444 | ,477 |  | ,000 |
| AodiaphDist | Pearson Correlation | ,043 | ,042 | ,032 | ,039 | ,188 | ,282 | ,091 | ,144 | .944^**^ | 1 |
|  | Sig. (2-tailed) | ,781 | ,785 | ,835 | ,799 | ,215 | ,060 | ,551 | ,345 | ,000 |  |
| EDV | Pearson Correlation | -.368^*^ | -,112 | ,087 | .374^*^ | ,069 | ,047 | -,122 | -,147 | ,054 | ,028 |
|  | Sig. (2-tailed) | ,014 | ,469 | ,575 | ,012 | ,656 | ,764 | ,430 | ,341 | ,729 | ,858 |
| EF | Pearson Correlation | ,092 | -,035 | ,203 | -,177 | -.333^*^ | -,288 | ,170 | ,188 | ,018 | ,043 |
|  | Sig. (2-tailed) | ,551 | ,823 | ,186 | ,250 | ,027 | ,058 | ,270 | ,222 | ,905 | ,780 |
| EDwallmass | Pearson Correlation | -,126 | -,131 | ,140 | ,084 | -,105 | -,121 | -,140 | -,163 | ,166 | ,138 |
|  | Sig. (2-tailed) | ,415 | ,396 | ,364 | ,587 | ,499 | ,435 | ,363 | ,289 | ,281 | ,371 |
| ESV | Pearson Correlation | -.328^*^ | -,015 | -,065 | .349^*^ | ,212 | ,174 | -,192 | -,217 | ,048 | ,009 |
|  | Sig. (2-tailed) | ,030 | ,925 | ,676 | ,020 | ,166 | ,258 | ,212 | ,157 | ,758 | ,956 |
| SV | Pearson Correlation | -.337^*^ | -,163 | ,153 | .349^*^ | -,008 | -,023 | -,061 | -,084 | ,042 | ,025 |
|  | Sig. (2-tailed) | ,025 | ,292 | ,323 | ,020 | ,959 | ,884 | ,694 | ,586 | ,786 | ,871 |
